# Multidimensional Proteomics Analysis of Amniotic Fluid to Provide Insight into the Mechanisms of Idiopathic Preterm Birth

**DOI:** 10.1371/journal.pone.0002049

**Published:** 2008-04-23

**Authors:** Irina A. Buhimschi, Guomao Zhao, Victor A. Rosenberg, Sonya Abdel-Razeq, Stephen Thung, Catalin S. Buhimschi

**Affiliations:** Department of Obstetrics, Gynecology and Reproductive Sciences, Yale University School of Medicine, New Haven, Connecticut, United States of America; Khon Kaen University, Thailand

## Abstract

**Background:**

Though recent advancement in proteomics has provided a novel perspective on several distinct pathogenetic mechanisms leading to preterm birth (inflammation, bleeding), the etiology of most preterm births still remains elusive. We conducted a multidimensional proteomic analysis of the amniotic fluid to identify pathways related to preterm birth in the absence of inflammation or bleeding.

**Methodology/Principal Findings:**

A proteomic fingerprint was generated from fresh amniotic fluid using surface-enhanced laser desorbtion ionization time of flight (SELDI-TOF) mass spectrometry in a total of 286 consecutive samples retrieved from women who presented with signs or symptoms of preterm labor or preterm premature rupture of the membranes. Inflammation and/or bleeding proteomic patterns were detected in 32% (92/286) of the SELDI tracings. In the remaining tracings, a hierarchical algorithm was applied based on descriptors quantifying similarity/dissimilarity among proteomic fingerprints. This allowed identification of a novel profile (***Q-profile***) based on the presence of 5 SELDI peaks in the 10–12.5 kDa mass area. Women displaying the ***Q-profile*** (mean±SD, gestational age: 25±4 weeks, n = 40) were more likely to deliver preterm despite expectant management in the context of intact membranes and normal amniotic fluid clinical results. Utilizing identification-centered proteomics techniques (fluorescence two-dimensional differential gel electrophoresis, robotic tryptic digestion and mass spectrometry) coupled with Protein ANalysis THrough Evolutionary Relationships (PANTHER) ontological classifications, we determined that in amniotic fluids with ***Q-profile*** the differentially expressed proteins are primarily involved in non-inflammatory biological processes such as protein metabolism, signal transduction and transport.

**Conclusion/Significance:**

Proteomic profiling of amniotic fluid coupled with non-hierarchical bioinformatics algorithms identified a subgroup of patients at risk for preterm birth in the absence of intra-amniotic inflammation or bleeding, suggesting a novel pathogenetic pathway leading to preterm birth. The altered proteins may offer opportunities for therapeutical intervention and future drug development to prevent prematurity.

## Introduction

Prematurity remains the leading cause of early neonatal deaths and long term neuro-developmental handicap [1], [2]. Despite extensive research and a variety of interventions and preventive strategies, the rate of preterm birth has steadily increased over the past 20 years to reach a high of 12.8 percent (%) in 2005 [3]. Several pathophysiological mechanisms have been implicated in triggering preterm birth [4]. Of these, genetic predisposition, fetal stress, excessive stretching, inflammation/ infection and decidual hemorrhage have received increased attention. [4]

In prior studies we concentrated our attention on investigating the changes in amniotic fluid proteome in the context of inflammation and decidual hemorrhage induced-preterm birth [5], [6] We devised a novel, stepwise strategy to extract relevant proteomic biomarkers based on filter preferences applied sequentially. This strategy was named “Mass restricted scoring or MR scoring” [5]. Based on these principles we defined relevant disease biomarker patterns characteristic for intra-amniotic inflammation and decidual bleeding [5], [6]. Proteomic identification techniques recognized the four protein biomarkers (which comprise the proteomic profile or MR score characteristic for intra-amniotic inflammation) as neutrophil defensin-2, neutrophil defensin-1, calgranulin C and calgranulin A. A combination of peaks corresponding to free hemoglobin (Hb) chains was component of the proteomic pattern characteristic for decidual hemorrhage [6]. In subsequent studies the presence of an inflammatory proteomic pattern in amniotic fluid proved to be predictive not only for preterm birth but also for histological funisitis and early onset neonatal sepsis [7], 8[]. Regrettably, notwithstanding recent advancements in comprehending the pathophysiology of preterm birth, the etiology of this syndrome still remains unknown [9].

The transition from the past era of hypothesis-driven research to that of a discovery-determined one stemmed from opportunities presented by the contemporaneous revolution in biomedical sciences such as the one recently witnessed in proteomics [10]. Proteomics seems to have all the attributes necessary to provide the needed breakthrough in understanding the physiopathology of idiopathic preterm birth and to identify novel inroads to help prevent its occurrence. To that end we expect that causes other that inflammation and bleeding would also generate aberrant amniotic fluid protein expression levels that translate in characteristic proteomic signatures related to the underlying cause. The objective of our study was to identify biomarker patterns relating to preterm birth in the absence of inflammation or bleeding and provide a comprehensive analysis of the amniotic fluid proteome in cases with such abnormal patterns.

## Methods

### Study population

We conducted a prospective study that included 285 women recruited following admission to the Labor and Birth unit or High Risk antepartum units at Yale New Haven Hospital (YNHH). Symptoms of preterm labor, preterm premature rupture of membranes (PPROM), advanced cervical dilatation (≥3 centimeters) and/or uterine contractions unresponsive to tocolysis were considered clinical indications for ultrasound-guided amniocentesis to rule-out infection. Women were enrolled consecutively based on a protocol approved by the Human Investigation Committee of Yale University. Written informed consent for the use of biological samples was obtained prior to the procedure. All women in this study had a singleton fetus at <37 weeks gestational age. Exclusion criteria included: viral infections (human immunodeficiency virus or hepatitis), anhydramnios and presence of fetal heart rate abnormalities (bradycardia, recurrent variable and late decelerations) requiring emergency intervention. Gestational age was established based on a second trimester ultrasonographic examination prior to 20 weeks in all instances. We defined preterm labor as presence of regular uterine contractions accompanied by cervical effacement and/or dilatation in patients <37 weeks gestational age. The diagnosis of PPROM was confirmed by vaginal amniotic fluid “pooling”, “nitrazine”, “ferning” or by an amniocentesis-dye positive test. Clinical chorioamnionitis was suspected in the presence of either fever >38°Celsius (C), uterine tenderness or fetal tachycardia. The decision to perform an amniocentesis and patient management including the decision to deliver the fetus were at the discretion of the clinical team independent of our study protocol. Patients received steroids for enhancement of fetal lung maturity if gestational age was between 24 and 32 weeks. Antibiotic therapy was initiated as per the usual PPROM protocol (ampicillin and erythromycin i.v. in the first 48 hours, followed thereafter by oral medication) [11]. After amniocentesis, all participants were followed prospectively to the point of delivery. We recorded whether preterm delivery of the fetus occurred subsequent to spontaneous onset of uterine contractions or secondary to a clinically indicated delivery aimed to deliver the fetus in the absence of uterine contractions (including gestational age >34 weeks, evidence of intra-amniotic infection, prolapsed cord, non-reassuring fetal heart rate) [12].

Following retrieval, amniotic fluid was cultured for aerobic and anaerobic bacteria, *Ureaplasma* and *Mycoplasma* species by the YNHH microbiology laboratory. The clinical laboratory tests performed for the purpose of diagnosing inflammation/infection were: glucose, lactate dehydrogenase (LDH), Gram stain, white (WBC) and red blood cell (RBC) counts. Research samples were spun at 3000×g at 4°C for 20 min., aliquoted and stored at –80°C for further study.

### Proteomic profiling for identification of patterns of intra-amniotic inflammation (MR score) and bleeding

**Figure 1:**
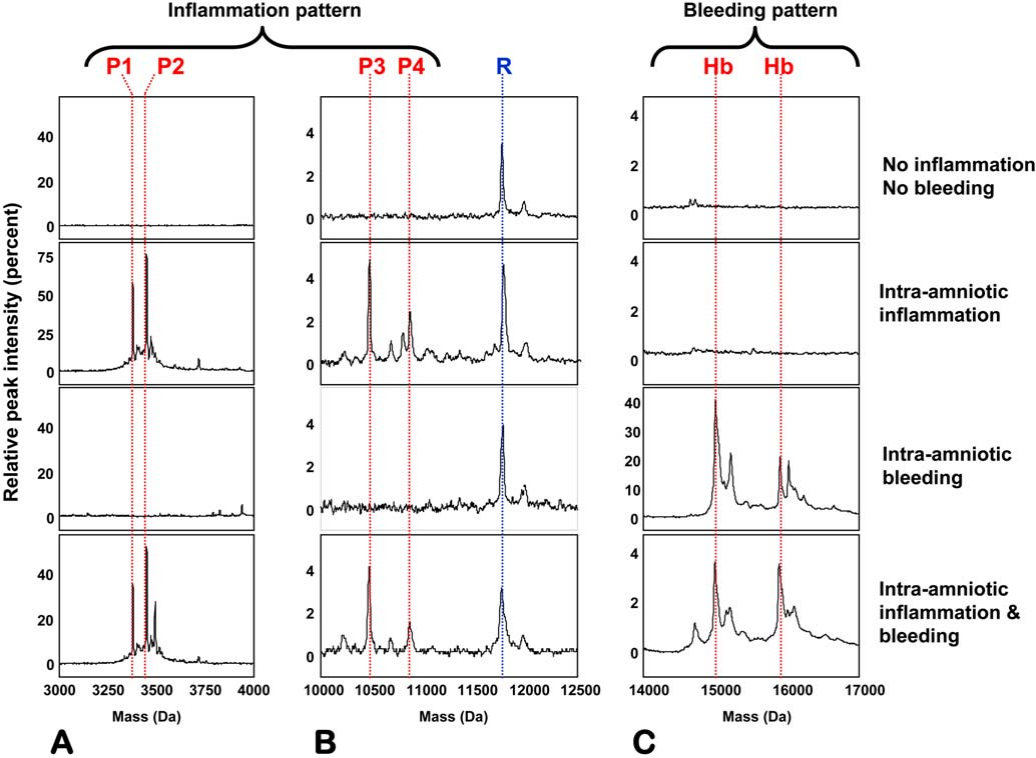
Proteomic patterns characteristic for intra-amniotic inflammation and bleeding. Three hypervariable areas of 4 representative SELDI tracings of amniotic fluid (A: 3–4 kDa, B: 10–12.5 kDa and C: 14–17 kDa) are shown. These areas contain the proteomic patterns characteristic of intra-amniotic inflammation (MR score composed of four protein biomarkers: P1: neutrophil defensin-2: P2: neutrophil defensin-1: 3448.09 Da, P3: calgranulin C, P4: calgranulin A), intra-amniotic bleeding (Hb chains) or both (bottom tracins). The x-axis of the tracings represents the molecular weight in Daltons; the y-axis represents the normalized peak intensity. R denotes a reference protein peak present in all fluid samples which corresponds to a fragment of beta-2 microglobulin.

We used surface-enhanced laser desorbtion ionization time-of-flight (SELDI-TOF) mass spectrometry to screen for the presence of biomarkers characteristic of inflammation and bleeding in fresh samples of amniotic fluid. The results were not made available to the clinical management team. The methodology for generation of the MR score and identification of bleeding biomarkers has been previously described [5], [6]. Briefly, 5 microliters (µL) of 10-fold diluted amniotic fluid in phosphate-buffered saline solution (PBS) was placed on spots of duplicate H4 arrays (8-spot H4 array; Ciphergen Biosystems). After 1-hour incubation, the sample was aspirated, and the spots individually washed with 20% aqueous acetonitrile solution, air-dried, and covered with energy absorbing molecule (EAM) matrix solution of either 1-µL of 20% saturated α-cyano-4-hydroxycinnamic acid (CHCA) in 0.5% trifluoroacetic acid/50% acetonitrile on one array, or 2 sequential applications of 1-µL 50% saturated solution (in 0.5% trifluoroacetic acid/50% acetonitrile) of sinapinic acid (SPA) on the other. The proteomic arrays were read in the the ProteinChip Reader (model PBS II; Ciphergen Biosystems) using the ProteinChip software (version 3.0; Ciphergen Biosystems). For identification of proteomic profiles characteristic for inflammation (the MR score) and bleeding (Hb peaks), examination of SELDI tracings was targeted in 3 mass ranges: 3000 to 4000 Daltons (Da) (CHCA), 10 to 12.5 kiloDaltons (kDa) (SPA), 14 to 17 kDa (SPA). The data for the biomarkers of the MR score were extracted from the 3000 to 4000 Da (P1 and P2, Figure 1A) and 10 to 12.5 kDa (P3 and P4, Figure 1B) ranges. The data for the Hb peaks were extracted from the 14 to 17 kDa region (Figure 1C). Both the MR score and Hb profile provide qualitative information regarding the presence or absence of intra-amniotic inflammation or bleeding, respectively [5], [6]. A categorical value of 1 is assigned if a biomarker peak is present and 0 if absent. Thus, the MR score (inflammation) ranges from 0 to 4, depending upon the presence or absence of each of the four protein biomarkers and is a measure of the severity of intra-amniotic inflammation. MR scores 3–4 are indicative of severe inflammation. The Hb score (ranging from 0–2 depending with the number of free Hb chains identified) indicates presence of intra-amniotic bleeding for any value different than 0 [6]. The generation of the MR and Hb scores for each SELDI tracing was performed by an investigator who was blinded to the origins of the amniotic fluid sample, clinical presentation, clinical outcome or results of the placental histological examination.

### Extraction of the proteomic pattern characteristic of idiopathic preterm birth

**Figure 2:**
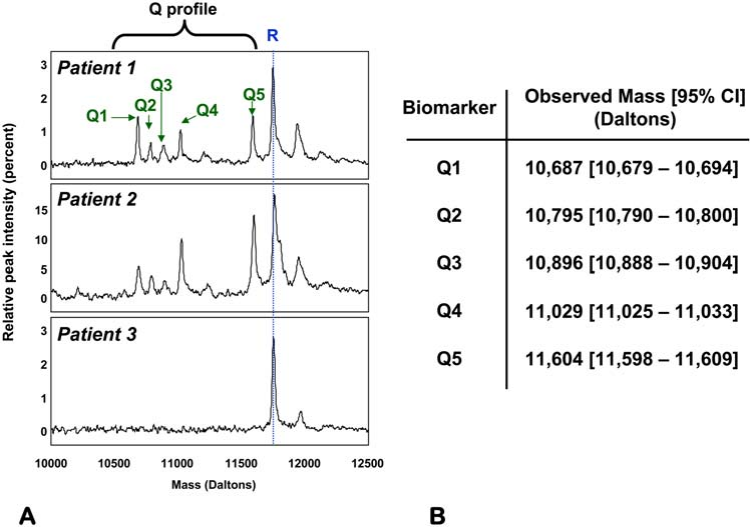
Biomarker peaks in the 10–12.5 kDa hypervariable region (*Q-profile*). A: Five SELDI peaks (Q1–Q5 denoted by green arrows) in the 10–12.5 kDa mass region appeared in a subgroup of the SELDI tracings analyzed (Patients 1 and 2 are examples). The third tracing (Patient 3) shows the lack of biomarkers in a woman that delivered at term. R denotes a reference protein peak present in all fluid samples which corresponds to a fragment of beta-2 microglobulin. B: experimental masses (average and 95% confidence interval (95%CI) for the five biomarker components of the *Q-profile*.

A novel computational algorithm was employed to analyze the proteomic information between 500 to 300,000 Da in our experimental conditions. From a bioinformatics perspective, SELDI tracings display silent areas alternating with hypervariable areas. Silent areas are mass ranges devoid of proteomic information and are identified by their similarity with the tracings obtained with the PBS diluent alone. In contrast, hypervariable areas are the mass ranges rich in potential biomarker peaks and are delineated by the high level of dissimilarity from the PBS tracings. For the purpose of reducing complexity, our algorithm first eliminated silent areas while extracting hypervariable areas. We next performed a hierarchical clustering of the tracings based on their similarity/dissimilarity in peaks present in hypervariable areas. Presence or absence of peaks was converted to binary strings with equal number of attributes and a hierarchical clustering algorithm applied. From the several hypervariable areas analyzed, we focused our attention on the mass area between 10 to 12.5 kDa due to the non-random clustering of the tracings based on a complex of five peaks in this region which we named the ***Q-profile*** (Figure 2A). The peak components are labeled Q1–Q5.

### Fluorescence two-dimensional differential gel electrophoresis (2D-DIGE)

To clarify and expand the biological significance of the ***Q-profile*** on a larger scale we employed 2D-DIGE on select pairs of amniotic fluid samples matched for maternal age, parity, gestational age at amniocentesis and membrane status. We used amniotic fluid samples selected from patients with the ***Q-profile*** who delivered preterm (n = 3) and compared the results with samples of amniotic fluid retrieved from women who presented with symptoms of preterm labor but delivered at term (CRL, n = 3). 2D-DIGE was performed using the Ettan DIGE system (GE Healthcare). Amniotic fluid proteins (67 µg) were labeled with either Cy3 (***Q-profile***) or Cy5 (CRL). A reference pool (25 µg total protein) was labeled with Cy2 and used as internal control. For each pair, labeled samples were pooled and isoelectric focusing was performed using an isoelectric point range of 3–10. SDS-PAGE on a 12% gel was performed for the second dimension. After spot detection, automatic background correction, spot volume normalization and volume ratio calculation, dye ratios were determined using DeCyder software (GE Healthcare). Spots corresponding to ≥1.5-fold changes [13] were robotically excised using the Ettan Spot Picker instrument (GE Healthcare) and subjected to automated in-gel tryptic digestion on the Ettan TA Digester (GE Healthcare). Automated MALDI-MS/MS spectra were acquired on the 4800 TOF/TOF proteomics analyzer (Applied Biosystems, Foster City, CA) and the resulting peptide masses subjected to database searching using Mascot algorithms (Matrix Science, Boston, MA). The remaining aliquots of digests of protein spots that were not identified by this approach were subjected to LC/MS/MS analysis (Micromass Q-TOF) followed by Sequest (Thermo Finnegan, San Jose CA) database searches.

### Pathway analysis

Differentially expressed proteins were classified based on the PANTHER (Protein ANalysis THrough Evolutionary Relationships) system (http://www.pantherdb.org) [14]. PANTHER is a unique resource that classifies genes and proteins by their functions, using published scientific experimental evidence and evolutionary relationships abstracted by curators with the goal of predicting function even in the absence of direct experimental evidence. Proteins are classified into families and subfamilies of shared function, which are then categorized using a highly controlled vocabulary (ontology terms) by biological process, molecular function and molecular pathway.

### Histological evaluation of the placenta

The diagnosis of histological chorioamnionitis was based on neutrophil infiltration of the chorionic plate, amniochorionic membranes and chorio-decidua. Three histological stages of chorioamnionitis [15] (stage I: intervillositis, stage II: chorionic inflammation, and stage III: full-thickness inflammation of both chorion and amnion) were complemented by the histological grading system devised by Salafia et. al. which includes four grades of inflammation of the amnion, chorion-decidua and funistis [16]. Presence of hemosiderin in the placental basal plate, extraplacental membranes and decidua confirmed by iron stains was suggestive of bleeding [17].

### Immunoassays for interleukin-6 (IL-6) and matrix metalloprotease-8 (MMP-8)

ELISA for human IL-6 (Pierce-Endogen, Rockford, IL) and MMP-8 (R&D Systems, Minneapolis, MN) were performed in duplicate by investigators unaware of clinical outcomes. Amniotic fluid samples were diluted to allow for appropriate interpolation of the results. The minimal detectable concentration for IL-6 was 1-picogram/milliliter and less than 0.02-nanogram/milliliter for MMP-8. The inter- and intra-assay coefficients of variation were <10% for IL-6 and <6% for MMP-8, respectively.

### Statistical analysis

Data is presented as mean and standard deviation (SD) or, if non-normally distributed, as median and range. Statistical analyses were performed with Sigma Stat, version 2.03 (SPSS Inc., Chicago, IL). Data was compared with one-way ANOVA followed by Student Newman Keuls tests (parametric) or Kruskal-Wallis on ranks followed by Dunn's tests (non-parametric), to adjust for multiple comparisons as appropriate. Comparisons between proportions were done with Chi-square or Fisher's exact tests. A *p* value of <0.05 was considered significant.

## Results

### Description of a novel proteomic pattern (Q-profile) associated with preterm birth in the absence of inflammation and bleeding

**Figure 3:**
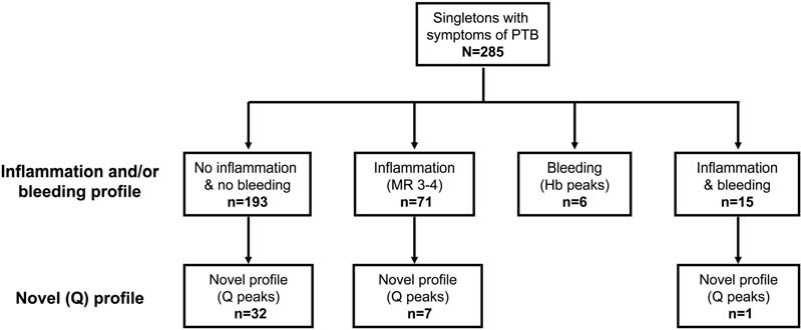
Distribution of amniotic SELDI tracings based on proteomic patterns.

Based on the established proteomic fingerprints of inflammation and bleeding, we identified that of the 285 SELDI tracings screened, 92 (32%) had either evidence of intra-amniotic inflammation (n = 71, MR 3-4), bleeding (n = 6, Hb peaks present) or both (n = 15) (Figure 3). Application of the hierarchical algorithm to the hypervariable areas of the remaining 193 tracings (no inflammation and no bleeding patterns) identified a novel discriminatory profile consisting of 5 proteomic peaks in the 10–12.5 kDa area of interest (***Q-profile***) (Figure 2A) in 32 patients. In addition, peaks corresponding to those of the ***Q-profile*** were found in 7 cases that also had inflammation and in one case where bleeding coexisted with inflammation. The masses of the biomarkers components of the ***Q-profile*** are listed in Figure 2B.

### Clinical and outcome characteristics of women displaying the novel proteomic pattern (Q-profile)

The cases displaying any type of amniotic fluid proteomic fingerprint (n = 124) had a significantly higher frequency of preterm delivery (88%) compared to the rest (65%, p<0.001). To further understand the biological significance of the ***Q-profile*** we analyzed the cases that exclusively displayed this profile (n = 32) in comparison to four additional groups: those displaying the inflammation fingerprint alone (n = 64), the bleeding fingerprint alone (n = 6), the combined inflammation and bleeding profile (n = 14) and those displaying no proteomic profile who delivered at term (n = 33). In Table 1 we present the demographical and outcome characteristics of these 5 groups of women. As mentioned in *Methods* section, all women had clinical signs and symptoms of preterm birth at the time of amniocentesis. Women displaying either the ***Q-profile*** or the inflammation profile were at an earlier gestational age at time of amniocentesis compared to the reference group (ANOVA, p≤0.001), but there were no differences among the 4 groups that displayed any proteomic profile. Women with the ***Q-profile*** presented more often with intact membranes compared to the inflammation and/or bleeding groups. Among the groups with abnormal proteomic profiles, there was no significant difference in gestational age at birth. However, a significantly higher proportion of women displaying the bleeding and/or inflammation profile, underwent an indicated preterm delivery subsequent to amniocentesis results. Therefore, despite their expectant management, 84% (27/32) of the women with the still ***Q-profile*** delivered prematurely. The amniocentesis-to-delivery interval was longer for women displaying the ***Q-profile*** as compared to those displaying the proteomic fingerprint characteristic for inflammation and/or bleeding. Moreover, newborns in the inflammation groups (with or without bleeding), but not those with the ***Q-profile*** had significantly lower Apgar scores compared to all other groups.

**Table 1:** Demographic and clinical characteristics of women displaying various proteomic fingerprints in amniotic fluid

Variable	Q profile	Inflammation	Bleeding	Inflammation & bleeding	None & term delivery	*P* value
	n = 32	n = 64	n = 6	n = 14	n = 33	
***Characteristics at amniocentesis***						
Age, *years* †	26 20–32	28 23–35	33 30–36	31 26–34	25 20–30	0.084
Gravidity †	2 1–3	2 1–4	3 2–3	3 2–3	2 1–3	0.754
Parity †	1 [0–1]	0 [0–1]	2 [0–2]	1 1–2	1 [0–1]	0.251
Non-Caucasian race ‡	20 (63)	47 (73)	2 (33)	9 (64)	24 (73)	0.279
History of preterm birth ‡	9 (27)	14 (35)	0 (0)	2 (14)	5 (16)	0.431
Gestational age, *weeks* §	25±4	27±4	26±6	27±4	30±4	<0.001
Ruptured membranes ‡	3 (9)	29 (45)	3 (50)	12 (86)	0 (0)	<0.001
Uterine contractions ‡	16 (50)	36 (56)	1 (17)	7 (50)	13 (42)	0.342
Clinical chorioamnionitis ‡	1 (3)	11 (17)	0 (0)	1 (7)	2 (6)	0.144
Cervical dilatation †	1 [0–3]	2 [0–4]	1 [0–4]	1 [0–2]	0 [0–2]	0.023
Tocolysis ‡	12 (38)	24 (38)	0 (0)	8 (57)	13 (39)	0.209
***Outcome characteristics***						
Amniocentesis-to-delivery, *days* †	40 [7–80]	1 [0–2]	2 1–8	0 [0–2]	55 [42–86]	<0.001
Gestational age at delivery, *weeks* †	33 26–35	27 24–31	31 23–33	28 25–31	39 37–39	<0.001
Term delivery ‡	5 (16)	0 (0)	0 (0)	0 (0)	33 (100)	<0.001
Indicated preterm delivery ‡	5 (16)	42 (66)	4 (67)	11 (79)	0 (0)	<0.001
Birthweight, *grams* †	2,160 [1,088–2,401]	1,030 [750–1,513]	1,430 [769–1,902]	1,138 [755–1,580]	3,100 [2,854–3,476]	<0.001
Cesarean delivery ‡	10 (31)	28 (44)	2 (33)	5 (36)	3 (39)	0.915
Apgar score at 1 minute †	8 6–9	5 2–8	9 4–9	7 3–8	9 8–9	<0.001
Apgar score at 5 minutes †	9 8–9	8 4–9	9 5–9	9 7–9	7 [9–8]	<0.001

† Data presented as median [interquartile range] and analyzed by Kruskal-Wallis ANOVA;

‡ Data presented as n (%) and analyzed by Chi square;

§ Data presented as mean±SD and analyzed by One Way ANOVA.

In Table 2 we present the results of the amniotic fluid analysis for the 5 different groups. We determined that the ***Q-profile*** group is characterized by amniotic fluid results similar to the group with no profile and delivery at term.^8^ All women displaying the ***Q-profile*** had negative Gram stain, negative culture results and normal levels of inflammatory cytokines. This was determined to be in sharp contrast to the women who had the inflammation profile present (with or without bleeding) who had significantly lower glucose and elevated LDH activity, WBC, RBC counts and elevated levels of IL-6 and MMP-8. The incidence of a positive Gram stain and/or amniotic fluid culture result was also significantly higher in the inflammation groups. The bleeding alone group was characterized by elevated LDH activity levels in the context of a higher RBC (but not WBC) count, and normal IL-6 and MMP-8 levels.

**Table 2:** Results of amniotic fluid analysis

Variable	Q profile	Inflammation	Bleeding	Inflammation & bleeding	None & term delivery	*P* value
	n = 32	n = 64	n = 6	n = 14	n = 33	
***Clinical laboratory analysis***						
Glucose, *mg/dL* †	30 23–36	7 2–18	17 7–28	4 2–13	34 [24–43]	<0.001
LDH, *U/L* †	155 [119–217]	619 [410–1475]	338 [171–1705]	456 [273–1312]	137 [102–171]	<0.001
WBC, *cells/mm^3^* †	3 1–5	700 [108–1,520]	10 1–21	755 [42–3750]	3 1–6	<0.001
RBC, *cells/mm^3^* †	8 [3–90]	167 [17–1,006]	110 [23–160,338]	8,450 [400–312,000]	18 [7–217]	<0.001
Positive Gram stain ‡	0 (0)	27 (43)	1 (17)	5 (36)	0 (0)	<0.001
Positive cultures ‡	0 (0)	40 (63)	1 (17)	8 (57)	0 (0)	<0.001
***Immunoassays***						
IL-6, *ng/mL* †	0.5 [0.2–1.2]	14.1 [6.0–43.8]	2.9 [1.3–4.8]	9.1 [5.5–15.9]	0.2 [0.1–0.2]	<0.001
MMP-8 *ng/mL* †	12.0 [3.9–22.4]	413.9 [178.5–1,087.5]	54.8 [19.2–68.9]	465.5 [75.2–411.9]	4.0 [2.7–5.6]	<0.001

† Data presented as median [interquartile range] and analyzed by Kruskal-Wallis ANOVA.

‡ Data presented as n (%) and analyzed by Chi square.

The results of the pathologic examination of the placenta were available in 101 of the cases in the 5 groups (Table 3). Overall, the prevalence of histological chorioamnionitis (stages II-III) was 65% (65/101). Similar to the group of women who delivered at term, the women with the ***Q-profile*** had virtually no evidence of chorioamnionitis, amnionitis or funisitis, despite delivering preterm. Lastly, bleeding was more frequently confirmed histologically in the context of bleeding-induced preterm birth (with and without inflammation).

**Table 3:** Results of histological analysis of the chorionic plate, placental membranes and umbilical cord.

Variable	Q profile	Inflammation	Bleeding	Inflammation & bleeding	None & term delivery	*P* value
	n = 18	n = 58	n = 5	n = 14	n = 6	
Chorioamnionitis, *stages II-III* ‡	2 (11)	49 (84)	1 (20)	11 (79)	2 (33)	<0.001
Amnionitis, *grades 2*–*4* ‡	2 (11)	43 (74)	1 (20)	10 (72)	1 (17)	<0.001
Choriodeciduitis, *grades 2*–*4* ‡	7 (39)	54 (93)	1 (20)	12 (86)	2 (33)	<0.001
Funistits, *grades 1*–*4* ‡	3 (15)	40 (69)	1 (20)	4 (31)	1 (17)	<0.001
Hemosiderin deposits present ‡	1 (6)	10 (17)	2 (40)	5 (38)	0 (0)	<0.001

‡ Data presented as n (%) and analyzed by Chi square.

Based on these results we conclude that women displaying the amniotic fluid ***Q-profile*** present most often with intact membranes and are managed expectantly in the context of normal amniotic fluid results. Yet, this group has a significantly higher risk for preterm birth which typically occurs in the context of normal placental histology. These findings concur with the concept that the ***Q-profile*** is representative of an amniotic fluid proteomic fingerprint which is different from that of the women with intra-amniotic inflammation or bleeding. No clinical or biochemical tests other than proteomic profiling of the amniotic fluid was able to delineate this high-risk group of women.

### Characterization of the amniotic fluid proteome representative of women displaying the novel profile (Q-profile)

**Figure 4:**
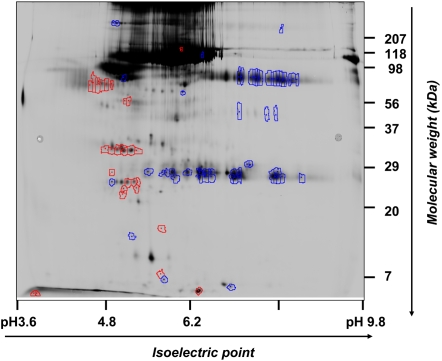
Representative two color merged 2D-DIGE image with up or down-regulated proteins selected for identification. Proteins in amniotic fluid with *Q-profile* present were labeled with Cy3 and proteins in control fluids with Cy5. Spots differentially regulated (≥1.5 fold in log spot volume) were selected for identification. Spots up-regulated are circled in red and spots down-regulated are circled in blue.

**Figure 5:**
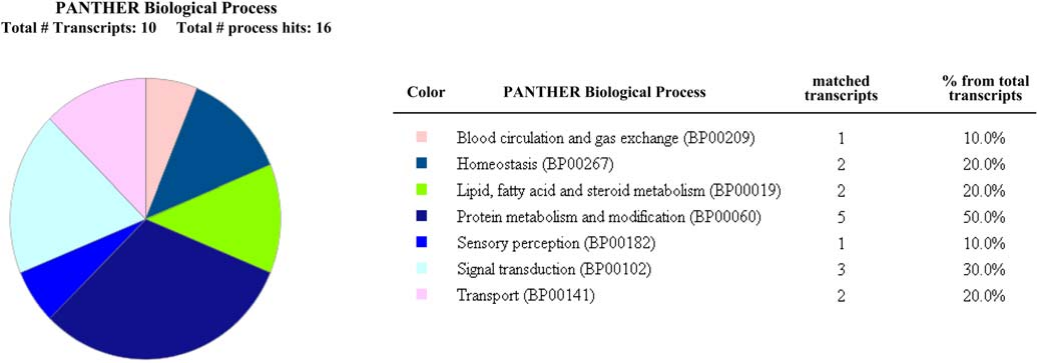
Classification of the amniotic fluid proteome dys-regulated in samples expressing the *Q-profile* using the PANTHER biological process classification system.

Figure 4 shows a representative 2D-DIGE image of proteins differentially expressed (red: spots up-regulated ≥1.5-fold, blue: spots down-regulated≥1.5-fold) between samples displaying the ***Q-profile*** and reference samples amniotic fluid samples (matched for gestational age at time of amniocentesis). Interestingly, more spots (n = 60) appeared down-regulated in the amniotic fluids displaying the ***Q-profile*** rather than up-regulated (n = 28). Of the spots picked for identification, 22 matched to a protein in the database with more than one peptide. Few spots matched to the same NCBI GenID transcript number. In fact, a total of 17 distinct protein database matches appeared and were differentially regulated at least 1.5-fold (Table 4) among the 2 groups. Further data mining using the PANTHER classification system revealed that of the unambiguous identities, 10 protein products belonged to 7 distinct and well-classified biological processes. The most populated was protein metabolism (5/10 transcripts matched) and signal transduction (3/10 transcripts matched) (Figure 5). PANTHER further converged the aberrantly expressed identities by matching several transcripts to the same protein precursor in the database (Table 5). This allowed us to conclude that the amniotic fluids with ***Q-profile*** are characterized by up-regulation in insulin growth factor binding protein (IGFBP-1), apolipoproteins (APO A-I and APO A-IV), lumican and bikunin (alpha-1-microglobulin light chain protein, complex-forming glycoprotein heterogeneous in charge) and down-regulation in the anti-proteolytic factors alpha-1-antitrypsin (SERPINA1), alpha-2-antiplasmin (SEPRPINF1, pigment epithelium derived factor) (Table 5).

**Table 4:** Differentially expressed proteins in amniotic fluid samples displaying the *Q profile*. Results of MS analysis

NCBI GenID	Protein Identified	Matched spots	Search Score	Percent Coverage	Peptides matched	Fold change
***Up-regulated***						
gi|90108666	Chain C, Crystal Structure Of Lipid-Free Human Apolipoprotein A-I	2	327	82	19	3.8
gi|223373	complex-forming glycoprotein heterogeneous in charge (bikunin)	2	128	51	8	2.0
gi|61744449	insulin-like growth factor binding protein 1 isoform b precursor	3	181	33	6	2.0
gi|178779	apolipoprotein A-IV precursor	1	193	52	16	1.9
gi|642534	lumican	1	131	19	6	1.9
gi|4505047	lumican precursor	1	127	22	7	1.9
***Down-regulated***						
gi|52695899	Chain L, Crystal Structure Of The Fab Yads1 Complexed With H-Vegf	1	121	50	6	5.1
gi|7438712	Ig kappa chain NIG93 precursor-human	2	149	48	6	3.1
gi|34526394	unnamed protein product	1	90	29	5	2.9
gi|34531791	unnamed protein product	1	126	35	12	2.5
gi|10835794	Chain C, Crystal Structure Of The Fab Fragment Of A Human Monoclonal Igm Cold Agglutinin	1	167	58	7	2.4
gi|5360679	anti-Entamoeba histolytica immunoglobulin kappa light chain	1	152	48	6	2.3
gi|29726975	Chain B, G-2 Glycovariant Of Human Igg Fc Bound To Minimized Version Of Protein A Called Z34c	1	117	58	8	2.0
gi|1942629	Alpha1-Antitrypsin	1	216	55	17	1.8
gi|55641313	PREDICTED: similar to Serine (or cysteine) proteinase inhibitor, clade A (alpha-1 antiproteinase, a (alpha1-antitrypsin)	1	116	39	16	1.7
gi|1144299	pigment epithelium-derived factor	1	83	26	7	1.7
gi|15825648	Chain K, Crystal Structure Of The Intact Human Igg B12 With Broad And Potent Activity Against Primary HIV-1 Isolates	1	102	36	11	1.5

**Table 5:** Classification and convergence of identified transcripts into PANTHER ontologies

Protein IDs	PANTHER Best Hit	PANTHER Biological Process	PANTHER Pathway	PANTHER Molecular Function
***Up-regulated***				
NP_001013047	INSULIN-LIKE GROWTH FACTOR	Signal transduction (ECM protein-mediated)	PI3 kinase	Miscellaneous function
NP_000587	BINDING PROTEIN 1	Homeostasis (growth factor)		
NP_000030	APOLIPOPROTEIN A-I	Lipid and fatty acid transport	–	Transporter
		Transport		Apolipoprotein
NP_000473	APOLIPOPROTEIN A-IV	Lipid and fatty acid transport	–	Transporter
		Transport		Apolipoprotein
		Blood circulation and gas exchange		
NP_002336	LUMICAN	Signal transduction (cell adhesion-mediated)	–	Receptor
		Sensory perception (vision)		ECM
NP_001624	ALPHA-1-MICROGLOBULIN/BIKUNIN	Protein metabolism (proteolysis)	–	Serine protease inhibitor
***Down-regulated***				
NP_001002235 NP_000286 NP_001002236	ALPHA-1-ANTITRYPSIN	Protein metabolism (proteolysis)	Coagulation	Serine protease inhibitor
NP_002606	ALPHA-2-ANTIPLASMIN	Protein metabolism (proteolysis)	Coagulation	Serine protease inhibitor

Abbreviations: ECM: extracellular matrix

## Discussion

Employing multidimensional proteomics analysis of amniotic fluid coupled with bioinformatic algorithms we were able to identify a novel subgroup of patients at risk for preterm birth that share common clinical, biochemical and proteomic features. These cases were distinct from those characterized by intra-amniotic inflammation or bleeding. The findings of the present study further support the notion that preterm birth is the result of several distinct pathogenetic pathways for which increased uterine contractility, cervical ripening and/or PPROM are simply common clinical features of the end product.

Proteomics encompasses protein separation and identification techniques along with the necessary bioinformatic tools that allow for biomarker discovery. Two current and rather opposing proteomic approaches have emerged. One relies on generating proteomic patterns from biological samples using high-throughput mass spectrometry approaches while minimizing the need to know the identity of the discriminatory biomarkers (proteomic pattern diagnostic approach) [18]. The other focuses on identification of proteins by digesting them into peptides and sequencing them using tandem mass spectrometry and database searching (identification-centered proteomic approach) [19]. In several prior studies, we and others have mined the amniotic fluid proteome for combinations of biomarkers that would separate pathogenetic pathways leading to preterm birth and could potentially be used as predictors of outcome [10] In the present study we used a multidimensional proteomic approach that combined SELDI-based diagnostic pattern proteomics with identification-based techniques (2D-DIGE) to find and understand changes in amniotic fluid proteome associated with spontaneous preterm birth in the absence of inflammation and bleeding. While in all prior studies [5], [6], [10] the relevant proteomic profiles were derived by comparing a “diseased set” versus a “normal set” as defined by other non-proteomic clinical, biochemical or microbiological criteria (differential proteomics), in the present study we used a reverse approach. Since none of the current tools available in clinical practice were of use to discriminate our patient pool despite significant differences in their pregnancy outcomes we sought to define a novel clinical instrument. After we eliminated the proteomic tracings characterized by diagnostic patterns indicative of the presence of inflammatory and bleeding, among the remaining cases we found a distinct cluster of patients characterized by presence of 5 SELDI peaks in the 10–12.5 kDa region (***Q-profile***). By exploring the common clinical characteristics of this subgroup we determined that these women presented most often with preterm labor and intact membranes in the context of normal amniotic fluid clinical and microbiological results. However, the majority of cases in this subgroup delivered prematurely despite tocolytic and expectant management per standard protocols. To our knowledge the ***Q-profile*** is the first proteomic pattern with the ability to identify women at risk for preterm birth in the absence of intra-amniotic inflammation and bleeding.

With few exceptions, the current stage of medical practice emphasizes disease classification based on shared clinical features which together are representative of a syndrome. Assignment of individual cases to these diagnostic categories frequently dictates treatment choices. However, while in clinical instances such as tumors or infectious etiologies the medical treatment is further conditioned based on strict histopathologic or microbiologic examinations, obstetrical practice lags seriously behind in this respect. Perhaps, our failure to recognize and prevent preterm birth rests with the inability to rapidly and accurately discern among the several distinct pathogenetic mechanisms leading to premature delivery. Personalized medicine, stands at the opposite end of this “one size fits all” approach and relies heavily on development of classification algorithms based on subclinical features and biomarkers [20]. Nowhere is a “pathway specific targeted treatment approach” more needed than in preventing premature delivery and its consequences to the newborn baby. To further identify novel pathways leading to preterm birth which may allow for individualized targeted treatment we proceeded with a proteomic identification-centered approach using the classical differential proteomics paradigm by defining the “disease set” as the samples expressing the ***Q-profile*** in the context of a preterm delivery. In the current study we have determined that the differentially expressed proteins which characterize the ***Q-profile*** are primarily involved in protein metabolism, signal transduction and transport. The identities of the proteins matched in the database pointed to a metabolic rather than inflammatory abnormality since none of the protein identities had apparent inflammatory function.

APO A-I and APO A-IV are associated with high density lipoprotein (HDL) and chylomicrons and have important roles in cholesterol, trygliceride and phospholipid transport and metabolism [21]. Most recently it has been proposed that APO A-IV is a satiety factor acting centrally in the brain. In fasted young animals there is a dramatic increase in liver mRNA synthesis and serum levels of APO A-I and A-IV in response to glucocorticoids and insulin increased by fasting stress [22]. APO A-I and A-IV have been described previously in amniotic fluid, but to our knowledge there has been no study investigating their levels or phenotypic variants as it relates to the fetus and to preterm birth [23].

IGFBP-1 is the predominant insulin-like growth factor binding protein in amniotic fluid [24]. It is produced by the decidua and fetal liver and it is thought to play an important role in fetal growth [25]. Higher mid-pregnancy levels of IGFBP-1 have been associated with growth failure [26]. Using an *in vitro* model Popovici et al demonstrated that hypoxia up-regulates fetal hepatocyte IGFBP-1 mRNA steady-state levels and protein, which is the major IGFBP derived from the fetal hepatocytes [27]. Another protein found differentially abundant in samples with the ***Q-profile*** was lumican, a member of the small leucine-rich proteoglycan family (SLRP) with important roles in embryonic development, tissue repair, tumor growth, organization of collagens [28] and maintenance of corneal transparency [29].

Bikunin (also known as the “complex-forming glycoprotein heterogeneous in charge”) is a Kunitz-type serin-protease inhibitor predominantly found in amniotic fluid and to a lesser degree in urine and serum where it circulates bound to the inter-alpha inhibitor proteins [30] A number of studies have shown that many serine protease inhibitors have complex effects on cellular growth and differentiation unrelated to their anti-proteolytic function [31] Bikunin in particular has the ability to modulate cell growth and to block cellular calcium uptake and is currently being evaluated as a novel anti-metastatic agent [32], [33]. Additionally, bikunin has a significant anti-inflammatory function by blocking systemic cytokine induction in response to endotoxin challenge *in vivo* [34] Finally, bikunin inhibits entotoxin-induced preterm labor in mice, [35] inhibits prostaglandin expression from amnion cells [36] suppresses uterine contractions [37] by reducing calcium influx in myometrial cells [38] and suppresses premature cervical ripening [39] which are all key pathogenetic processes necessary for preterm birth. The observed upregulation in bikunin levels may reflect an endogenous compensatory mechanism aimed to prolong pregnancy and downplay inflammatory processes. This may explain the longer duration to delivery in women with Q profile compared to the other groups exhibiting abnormal proteomic profiles, despite the similar gestational age at amniocentesis.

All proteins found differentially down-regulated in amniotic fluid expressing the ***Q-profile*** (pigment epithelium-derived factor [PEDF] and alpha-1 antitrypsin) were members of serpin superfamily. The roles of these factors in amniotic fluid are not well understood. PEDF is known to be the most potent inhibitor of angiogenesis in the mammalian ocular compartment [40]. It also has neurotrophic activity, both in the retina and in the central nervous system, and is highly up-regulated in young versus senescent fibroblasts [40]. No prior study investigated the presence PEDF in amniotic fluid or defined “normal levels”. One study found higher levels of trypsin activity and lower alpha 1-antitrypsin concentration in amniotic fluid of women with PPROM [41] but a subsequent study found no difference between amniotic fluid alpha-1 antitrypsin in premature birth with intact versus ruptured membranes or in those with intra-amniotic infection [42].

The present study demonstrates how diagnostic pattern proteomics can be used in conjunction with identification-centered proteomic techniques and bioinformatic tools to provide insight into a problem which has frustrated clinicians for decades. We acknowledge that at this time due to the limitation of the current technology we have not been able to identify the specific proteins responsible for the five SELDI peaks of the ***Q-profile***. Furthermore as SELDI and 2D-DIGE technologies are not equivalent, the biomarker peaks comprising the Q profile may not be part of the differentially expressed identities. Yet the SELDI diagnostic pattern itself proved essential in isolating a subgroup of preterm birth cases with unique features at the level of their amniotic fluid proteome. This will perhaps prompt a future research direction and paradigm into the prevention of prematurity.
